# On the Operation for Artificial Pupil

**Published:** 1815-03

**Authors:** John Stevenson

**Affiliations:** Great Russel-street, Bloomsbury-square


*For the Medical and Physical Journal.*

### *On the Operation for Artificial Pupil;* by Mr. STEVENSON.

THE manner in which you have been pleased to notice, among the articles of medical intelligence in the last Number of your long-established Journal, the success with which I performed, in two instances, the operation for artificial pupil, suggested to me by the celebrated inventor Dr. Assalini, seems to require a few words, lest I should be suspected of withholding from the public any important practical improvement, the result of my own observation and experience. In the present stage of the business, I have availed myself of the directions liberally imparted to me by a distinguished foreign practitioner. As, however, the process seems to possess a decided superiority over every other mode hitherto recommended for the same purpose, I should feel a satisfaction in detailing the particulars, assigning, at the same time, my reasons for giving it a preference; but, by so doing, I should forestal the Professor's valuable work on this subject, written in the Italian language, and of which a translation is now preparing for the press, under the able superintendance of Mr. Pettigrew. When the performance shall have made its appearance in an English dress, will be the proper time to suggest any alteration which frequently repeated practice may suggest, and the advantages of which can be only proved by experience.

JOHN STEVENSON.

*Great Russel-street, Bloomsbury-square;*
*Jan.* 22, 1815.

